# Impact of a structured review session on medical student psychiatry subject examination performance

**DOI:** 10.12688/f1000research.7091.2

**Published:** 2015-10-14

**Authors:** Shan H. Siddiqi, Kevin J. Black, Fay Y. Womer

**Affiliations:** 1 Department of Psychiatry, Washington University School of Medicine, St. Louis, MO, USA; 2 Departments of Neurology, Radiology, and Anatomy & Neurobiology, Washington University School of Medicine, St. Louis, MO, USA

**Keywords:** Medical education, NBME, shelf, review session, medical students, academic psychiatry, clinical clerkship

## Abstract

**Introduction: **

The National Board of Medical Examiners (NBME) subject examinations are used as a standardized metric for performance in required clerkships for third-year medical students. While several medical schools have implemented a review session to help consolidate knowledge acquired during the clerkship, the effects of such an intervention are not yet well-established. An improvement in NBME psychiatry examination scores has previously been reported with a single end-of-clerkship review session, but this was limited by a small sample size and the fact that attendance at the review session was optional, leading to likely selection bias.

**Methods: **

A 1.5-hour structured review session was conducted for medical students in the last week of each 4-week psychiatry clerkship between September 2014 and July 2015. Students were required to attend unless excused due to scheduling conflicts. Scores on the NBME psychiatry subject exam were compared with those of students taking the examination in the corresponding time period in each of the previous two academic years.

**Results: **

83 students took the exam during the experimental period, while 176 took the exam during the control period. Statistically significant improvements were found in mean score (p=0.03), mean for the two lowest scores in each group (p<0.0007), and percentage of students scoring 70 or less (p=0.03). Percentage of students achieving the maximum possible score (99) was higher in the experimental group, but did not reach significance (p=0.06).

**Conclusions: **

An end-of-clerkship review session led to increased mean scores on the NBME psychiatry subject examination, particularly for students at the lower end of the score range. Future research should investigate the impact of such an intervention in other specialties and other institutions.

## Background

The National Board of Medical Examiners (NBME) subject examinations are widely used in North America as a means of assessing overall performance and potential need for remediation in required third-year medical student clerkships; their utility is rooted in the fact that they provide a standardized and objective measure of knowledge acquired during the clerkship ^1^. While the utility of NBME examinations for internal evaluation of students has been questioned ^2^, this notion is challenged by the findings that performance on these examinations is correlated with other measures of a medical student’s knowledge base ^3, 4^, suggesting that higher scores are associated with improved overall educational outcomes. Furthermore, these scores are also correlated with a student’s eventual performance on the United States Medical Licensing Exam (USMLE) Step 2CK, which is a critical component of evaluation for residency selection ^5^.

However, strategies for preparing students for NBME examinations remain inconsistent ^6^. This process is particularly challenging for the psychiatry subject examination (PSE), in which performance has been found to be more strongly associated with interpersonal skills than with subjective faculty evaluations of a student’s medical knowledge and clinical skills ^7^, although alternate measures of student performance (including faculty evaluations and standardized patient encounters) are still correlated with PSE scores ^8^.

The impact of structured teaching on PSE scores has garnered some attention in the literature. Prior studies have demonstrated a significant improvement with a series of eight resident-led tutorials ^9^ and with a complete curriculum overhaul with a goal of improving scores ^10^. A single end-of-clerkship review session for the subject examination has also demonstrated an increase in scores, but this study was limited by a relatively small sample size, which limited the range of outcomes that could be effectively measured, and by potential selection bias, since attendance at the session was not mandatory ^11^. We investigated the impact of a single review session with a larger sample size and with mandatory attendance.

## Methods

The study retrospectively investigated scores on the PSE after implementation of a review session covering a general overview of adult psychiatry with a focus on topics that are critical for medical students to understand. The review session was conducted less than 1 week before students were required to take the PSE. Students were required to attend, but were excused in the event of a conflict with their rotation schedules. Data were analyzed retrospectively based on de-identified scores provided by the NBME. This study was deemed exempt from review by the institutional review board at Washington University in St. Louis, which determined that consent from individual students was not required and students need not be notified because data were de-identified prior to retrospective analysis. The Associate Dean for Education at Washington University School of Medicine also approved the retrospective review of de-identified scores.

The review session was based on an interactive case-based discussion of evaluation and management of common psychiatric problems, with a focus on topics that are commonly misunderstood by medical students. The session was designed and conducted by a resident physician (SHS) with prior experience in developing study materials for various standardized examinations, including the PSE. Cases demonstrated hypothetical patients with mania, depression, psychosis, substance abuse, anxiety/panic, eating disorders, personality disorders, somatoform disorders, and psychotropic medication toxicity. Additional non-case-based discussions were included to differentiate the types of dementia and understand legal/ethical issues in psychiatry. Child psychiatry topics were not included because the clerkship already included a separate lecture on child psychiatry during the same week. Detailed psychopharmacology was also not included due to time constraints; instead, students were advised to independently review mechanisms, indications, and toxicity profiles of the different classes of psychotropic medications.

The experimental group consisted of nine groups of students completing their psychiatry clerkships between September 2014 and July 2015. The control group consisted of students completing the examinations during the corresponding time periods in the previous two academic years; the other months in previous years were not included to avoid confounding due to the tendency of scores to increase as the academic year progresses. No other changes were made to the students’ lecture schedules.

Statistical analyses were completed with R version 3.2.0 using individual de-identified scores that were provided by the NBME in paper form. Mean scores for the full September to July period were compared between the experimental group and the control group via two-tailed paired t-test. In order to evaluate the effects on students with weaker knowledge base, a paired t-test was also used to compare means for all students who achieved lowest two scores in each 4-week clerkship block between the experimental group and the control group. A one-tailed Z-test for proportions was used to compare the fraction of students scoring 99 (the maximum possible score) and the fraction of students scoring 70 or less (typically corresponding approximately to the 10th percentile in the national sample; our school considers this a failing exam score that must be remediated to earn credit for the psychiatry clerkship).

## Results

Medical student scores on the psychiatry NBME subject examination before and after institution of a mandatory review session Date: Month and year of test administration, associated with the group of students that completed the clerkship block in the previous four weeks;
NBME Form: ID number of the specific version of the NBME subject examination administered to the group (i.e. form 2010–2 was the second test of the series written in 2010); Scores: Scaled scores for each student taking the exam in the clerkship block. Click here for additional data file. Copyright: © 2015 Siddiqi SH et al. 2015 Data associated with the article are available under the terms of the Creative Commons Zero "No rights reserved" data waiver (CC0 1.0 Public domain dedication).

Eighty-three students took the exam during the experimental period, while 175 took the exam during the control period. Statistically significant improvements were found in the mean score, the two lowest scores in each group, and the fraction of students scoring 70 or less. Improvement in fraction of students achieving the maximum possible score (99) did not reach significance (p = 0.06). These results are summarized in Table 1.

**Table 1.** Student performance with and without the review session.

Measure	Without review session	With review session	p-value
**Mean scaled score**	85.3 (95% CI 84.0 - 86.6)	87.8 (95% CI 86.1 - 89.4)	0.03
**Mean for two lowest scores in** **each student group**	74.1 (95% CI 72.7 - 75.5)	78.7 (95% CI 76.3 - 81.0)	0.0007
**Students scoring 99**	7.4% (13/175)	13.2% (11/83)	0.06
**Students scoring ≤ 70**	4.0% (7/175)	0% (0/83)	0.03

## Discussion

Implementation of a mandatory end-of-clerkship review session was associated with improvements in mean scores on the PSE, particularly for students whose scores were in the lower range. While similar improvements have been suggested in the past ^11^, this study reproduces these findings with a larger sample size, thereby allowing analysis of performance in different scoring ranges. This study also demonstrated a significant effect of the intervention despite higher baseline scores in this sample (mean baseline scaled score 85.3, compared to 77.2 in the previous study). Furthermore, attendance at the review session in this study was mandatory, thereby controlling for the selection bias introduced by the possibility that students choosing to attend a voluntary review session may have been more motivated at baseline.

Due to the retrospective nature of the analysis and lack of randomization, this study is subject to several limitations. Performance was compared between different academic years, so inter-class differences unrelated to the intervention may have confounded the results. Furthermore, while the review sessions followed a standardized format, we do not know how reproducible they may be in other academic settings.

This study did not investigate whether the improvement in students’ PSE performance translated to improvements in clinical skills. However, a recent large meta-analysis showed that clerkship grades (which usually incorporate NBME subject examination scores ^1^) and USMLE Step 2CK scores (which are correlated with NBME subject examination scores) predict a resident’s performance on both objective and subjective evaluations ^12^. Further research is needed in order to determine whether an end-of-clerkship review session translates to improvements in other measures of a student’s clinical skills and knowledge.

Overall, these results provide support for the notion that a single end-of-clerkship review session improves scores on the NBME psychiatry subject examination, even when eliminating selection bias by making the review session mandatory. Future studies should be geared at reproducing these findings in other specialties and standardizing the course for improved generalizability.

## Data availability

The data referenced by this article are under copyright with the following copyright statement: Copyright: © 2015 Siddiqi SH et al.

Data associated with the article are available under the terms of the Creative Commons Zero "No rights reserved" data waiver (CC0 1.0 Public domain dedication). 

*F1000Research*: Dataset 1. Medical student scores on the psychiatry NBME subject examination before and after institution of a mandatory review session, 10.5256/f1000research.7091.d102704.
