# Supplementary material for: Impact of a structured review session on medical student psychiatry subject examination performance
Source: F1000Res. 2015 Oct 14;4:897. Originally published 2015 Sep 24. [Version 2] doi: 10.12688/f1000research.7091.2 (PMC4648197; doi:10.12688/f1000research.7091.2)
Supplement: Peer review history

# Peer review: Referee response for version 1

**DOI:** 10.5256/f1000research.7635.r10688

**Published:** 2015-10-06

**Authors:** Andrew Lee

**Affiliations:** 1 Department of Ophthalmology, University of Iowa Hospitals and Clinics, Iowa City, IA, USA

In many institutions, the National Board of Medical Examiners (NBME) subject examinations are used as a performance metric for medical students and this study was conducted to see if a structured review session might improve scores on the exam.

The authors used a mandatory 1.5-hour structured review session in the last week of each 4-week psychiatry clerkship (September 2014 and July 2015) and the primary outcome measure was a comparison of scores on the NBME psychiatry subject exam before and after the interventional period.

The sample size was reasonable for a study of this type with 83 students in the experimental period and 176 in the historical control period. As might be expected, there were statistically significant improvements in mean score (p=0.03), mean for the two lowest scores in each group (p<0.0007), and percentage of students scoring 70 or less (p=0.03) in the experimental, interventional arm. Interestingly, the percentage of students achieving the maximum possible score (99) was higher in the experimental group, but did not reach significance (p=0.06).

The authors concluded that "an end-of-clerkship review session led to increased mean scores on the NBME psychiatry subject examination, particularly for students at the lower end of the score range."

These results suggest what is intuitively known but deserves emphasis: 1) structured, scheduled, mandatory, review is helpful in improving performance on standardized testing of the same or similar core content; 2) timeliness and proximity of the review to the testing may improve performance in the short term but this study (and many similar study designs) do not speak to long term retention and sustainability; and 3) multiple barriers exist in the status quo that might limit implementation or generalizability including the usual suspects (time, money, resources).

I have read this submission. I believe that I have an appropriate level of expertise to confirm that it is of an acceptable scientific standard.

# Peer review: Referee response for version 1

**DOI:** 10.5256/f1000research.7635.r10461

**Published:** 2015-10-02

**Authors:** Janet Wale

**Affiliations:** 1 Cochrane Collaboration Consumer Network, Brunswick, VIC, Australia

This article reports on a study of the impact of a structured review session at the end of a 4-week clerkship for third year medical students on psychiatry examination performance, over a 10-month period. Examination scores are compared with historical controls over the same period of time in the previous 2 years. Significant improvements were reported for overall scores and the lowest scoring students. The intervention appeared to be of value. The authors recommend further research for other medical specialties and in other institutions. This appears justified. The differences in scores were small but significant.

No ethical approval was required for the present small study but may be needed if more intricate study designs are used, including randomisation of larger numbers possibly through the use of cluster randomisation. The authors could provide some suggestions. They address the limitations in the present study well.

Some small corrections:

- The abstract in its introduction refers to "One prior study" and its finding - this could be written in more general terms....
- In the background (p3), 2nd para, line 1: ...for THESE examinations.... - not clear which examinations
- Methods (p3), right-hand column, 3rd para down, line 1: ...USING...USING - replace 1st with 'WITH'; line 2: ...that WERE provided by...
- Discussion (p4), right-hand column, top of text: ...these results provide [further] support...ie delete 'further'

I have read this submission. I believe that I have an appropriate level of expertise to confirm that it is of an acceptable scientific standard.
